# Crystal structure of an FIV/HIV chimeric protease complexed with the broad-based inhibitor, TL-3

**DOI:** 10.1186/1742-4690-4-1

**Published:** 2007-01-09

**Authors:** Holly Heaslet, Ying-Chuan Lin, Karen Tam, Bruce E Torbett, John H Elder, C David Stout

**Affiliations:** 1 Pfizer Global Research & Development, 2800 Plymouth Rd., Ann Arbor, MI 48105, USA; 2 Department of Molecular Biology, The Scripps Research Institute, 10550 N. Torrey Pines Rd., La Jolla, CA 92037, USA; 3 Department of Molecular & Experimental Medicine, The Scripps Research Institute, 10550 N. Torrey Pines Rd., La Jolla, CA 92037, USA

## Abstract

We have obtained the 1.7 Å crystal structure of FIV protease (PR) in which 12 critical residues around the active site have been substituted with the structurally equivalent residues of HIV PR (12X FIV PR). The chimeric PR was crystallized in complex with the broad-based inhibitor TL-3, which inhibits wild type FIV and HIV PRs, as well as 12X FIV PR and several drug-resistant HIV mutants [1-4]. Biochemical analyses have demonstrated that TL-3 inhibits these PRs in the order HIV PR > 12X FIV PR > FIV PR, with K_i _values of 1.5 nM, 10 nM, and 41 nM, respectively [2-4]. Comparison of the crystal structures of the TL-3 complexes of 12X FIV and wild-typeFIV PR revealed theformation of additinal van der Waals interactions between the enzyme inhibitor in the mutant PR. The 12X FIV PR retained the hydrogen bonding interactions between residues in the flap regions and active site involving the enzyme and the TL-3 inhibitor in comparison to both FIV PR and HIV PR. However, the flap regions of the 12X FIV PR more closely resemble those of HIV PR, having gained several stabilizing intra-flap interactions not present in wild type FIV PR. These findings offer a structural explanation for the observed inhibitor/substrate binding properties of the chimeric PR.

## Background

Feline immunodeficiency virus (FIV), a member of the lentivirus family, is a useful model for developing intervention strategies against lentiviral infection [5-7]. We aim to better understand the molecular basis of HIV-1 and FIV protease (PR) substrate and inhibitor specificities in order to develop broad-spectrum protease inhibitors that will inhibit both wild type and drug-resistant proteases. This approach has led to the development of TL-3, an inhibitor that is capable of inhibiting FIV, SIV, HIV-1 and several HIV-1 drug-resistant strains *ex vivo *[1-3], and other potential inhibitors with broad efficacy [8-10]. FIV PR, like HIV-1 PR, is a homodimer, but each monomer is comprised of 116 amino acids, as opposed to 99 amino acids for HIV-1 PR. The structure of FIV PR has been determined and compared to that of HIV-1 PR [11-13]. FIV PR, particularly in the active core region, is very similar to HIV-1 PR but only shares 27 identical amino acids (23% identical at amino acid level) and exhibits distinct substrate and inhibitor specificity [11,14-17]. FIV and HIV-1 PR each prefer their own matrix-capsid (MA-CA) junction substrate and FIV PR prefers a longer substrate than HIV-1 PR. Current clinical drugs against HIV-1 PR are poor inhibitors for FIV PR, primarily due to a smaller S3 substrate binding site in FIV PR which restricts binding of these drugs [2,3].

FIV PR is responsible for processing the FIV Gag and Gag-Pol polyproteins into 10 individual functional proteins[18]. Although the overall order of proteins in the Gag-Pol polyprotein in FIV and HIV-1 is similar, distinctions are also evident. HIV-1 Gag-Pol has an additional small spacer protein, p1, between nucleocapsid (NC) and p6 while the equivalent region in FIV is a single p2 peptide. In addition, HIV-1 lacks dUTPase (DU), which is encoded between reverse transcriptase (RT) and integrase (IN) within the Pol polyprotein in FIV. FIV PR, similar to HIV-1 PR, regulates its own activity through autoproteolysis at 4 cleavage sites in PR [12].

In both HIV-1 and FIV, the sequence of Gag and Gag-Pol precursor processing is highly regulated and critical for producing mature viruses for infection and replication [4,19-21]. Thus, PR is an attractive target for development of antiretroviral drugs. Protease inhibitors have drastically slowed the progression of disease and reduced the mortality rate in HIV-1 infected patients [22-25]. However, the high error rate of reverse transcriptase (RT) and high levels of viral replication, combined with lack of adherence to medication regimens, have led to the development of drug-resistant strains. Additional strategies are therefore needed for drug design to target cross-resistant PR variants.

**Figure 1:**
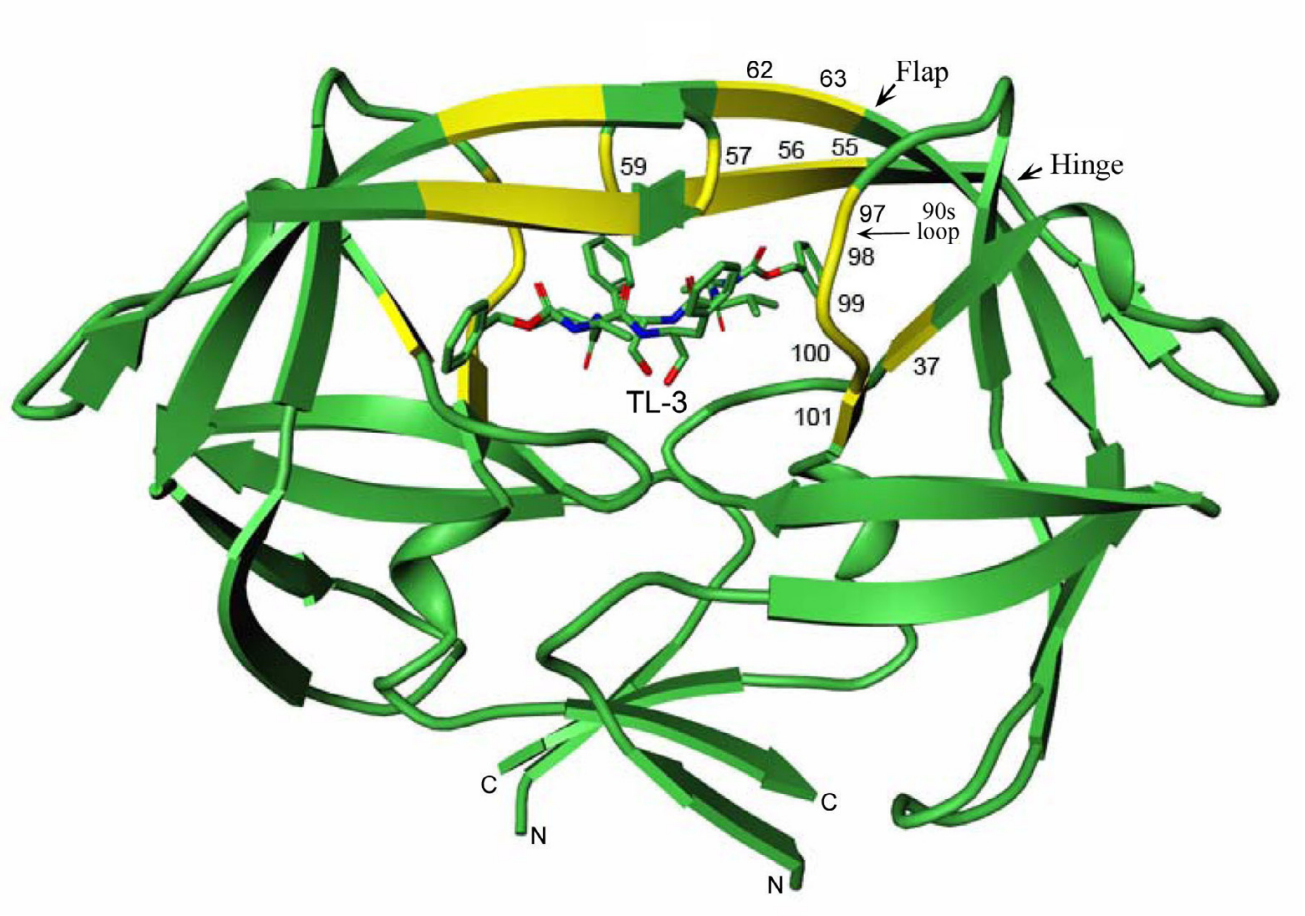
**Positions of mutation in chimeric 12X FIV protease**. The residues that were mutated to generate the 12X mutant of FIV protease are indicated in yellow. These included I37V in the active site core, N55M, M56I, I57G, V59I, G62F, and K63I in the flap region, and L97T, I98P, Q99V, P100N, and L101I in the "90s loop" region. The 2-fold axis of the 12X FIV protease dimer is vertical in the plane of the figure; the C_2 _axis of the bound inhibitor, TL-3, coincides with this 2-fold. All figures were generated using MoViT version 1.2.1 (Pfizer, La Jolla, CA, USA).

The properties of FIV PR and HIV-1 PR have been compared to better understand the molecular basis of retroviral PR substrate and inhibitor specificity. In previous studies, up to 24 amino acid residues in and around the active site of FIV PR were substituted at equivalent positions of HIV-1 PR and the specificity of mutant PRs was examined *in vitro *[2,4,15-17]. Substrate specificity of mutant FIV PRs was analyzed by examining cleavage efficiency on peptides representing HIV-1 and FIV cleavage sites. Inhibitor specificity of mutant PRs was assessed by measuring IC_50_/*K*_i _values of potent HIV-1 PR inhibitors. These experiments have revealed that some mutants, such as I37^32^V in the active core, N55^46^M, M56^47^I and V59^50^I in the flap region, and L97^80^T, I98^81^P, Q99^82^V, and P100^83^N, and L101^84^I in the "90s loop" region, retained comparable activity against FIV substrates while substantially changing substrate and inhibitor specificities toward that of HIV-1 PR (residue numbers for HIV PR indicated in superscript) (Fig. 1) [15,17]. Partial changes, both in inhibitor and substrate binding, were observed with over 40 chimeric PRs generated in the previous studies [4]. The most critical residues are embodied in a mutant containing 12 amino acid substitutions (referred to elsewhere as "12S FIV [4] and the studies reported here utilize this chimeric PR.

In order to better understand the molecular basis for the chimeric phenotypes described above, we have analyzed the crystal structure of a 12X FIV/HIV chimeric PR in complex with TL-3 and compared that structure to FIV and HIV wild type PRs in complex with the same inhibitor. The results show little alteration in the hydrogen bonding network formed between residues in the active site and flap regions of PR and the inhibitor. However, there is an increase in packing contacts formed between the P1 phenyl group of TL-3 and residues in the "90s loop" of the chimeric PR which involve 5 of the 12 mutations. These interactions help to explain the increase in potency of TL-3 against the 12X FIV PR relative to FIV PR. Additional mutations in 12X FIV PR localized to the flap regions of PR result in the formation of contacts within and between monomers, which may be related to changes in substrate processing efficiency.

## Results

### Two fold symmetric 12X FIV PR dimer binds C2 symmetric TL-3

To better understand the structural basis for the changes in substrate processing and efficiency as well as inhibitor specificity in the 12X FIV PR mutant, we determined the 1.7Å crystal structure of 12X FIV PR in complex with TL-3. The 12X FIV PR-TL3 complex crystallized in the space group *P*3_1_21 with a monomer in the asymmetric unit and the C_2 _axis of the protease dimer coincident with a crystallographic 2-fold (Table 1). As a result, the structure of the complex is an average of the two half-sites. Similarly, TL-3 was bound in the active site of the 12X FIV PR with its C_2 _axis of symmetry coincident with the crystallographic 2-fold and, therefore, was modeled as one half of the C_2 _symmetric compound.

**Figure 2:**
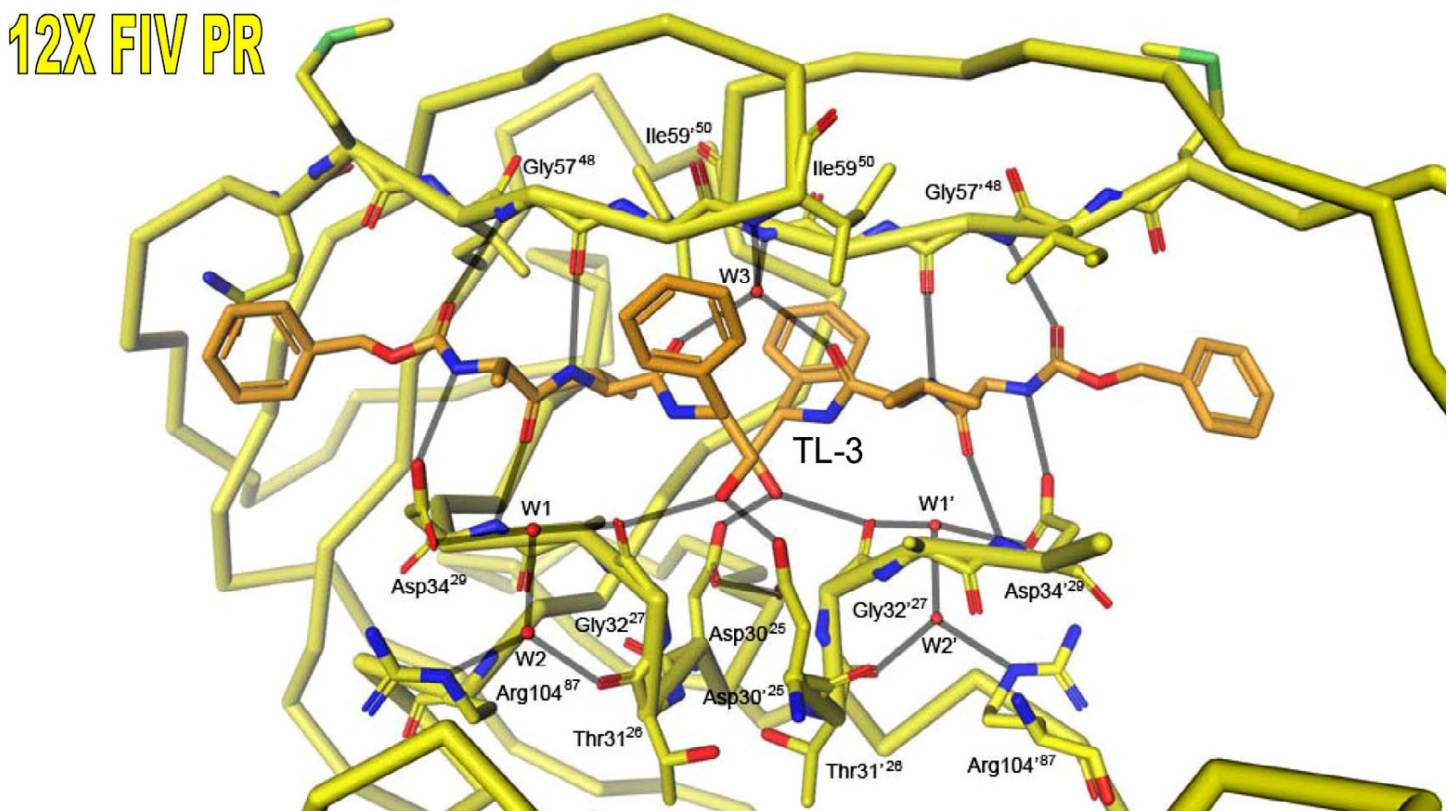
**Conformation of 12X FIV protease in complex with the inhibitor TL-3**. The hydrogen bonding network between TL-3 and 12X FIV protease is formed predominantly by main chain atoms of residues in the catalytic loop (residues 30–34) and flap regions (residues G57, I59) of the protease. The network is mediated by five ordered water molecules (W1–W3, W1'–W2'). This hydrogen bonding network is essentially identical to that formed by TL-3 in the active sites of both wild-type HIV and FIV protease [11, 12, 13, 26]. The equivalent residue numbers for HIV protease are indicated in superscript.

The network of hydrogen bonds between TL-3 and residues in the catalytic loop and flap region of the 12X FIV protease is essentially identical to that observed in the HIV PR-TL-3 and FIV PR-TL-3 complexes previously determined (Fig. 2) [13,26]. This hydrogen bonding network is mediated by four central water molecules and another coincident on the C_2 _axis, and includes the two pairs of hydrogen bonds that form critical interactions between the flap regions of the PR and the inhibitor. However, the 12X FIV PR complex lacks the water molecule which bridges the P4 carboxybenzyl group and Asp34^29 ^in the HIV PR-TL-3 complex [26].

### Mutations localized to 90s loop result in the formation of packing contacts with bound TL-3

**Figure 3:**
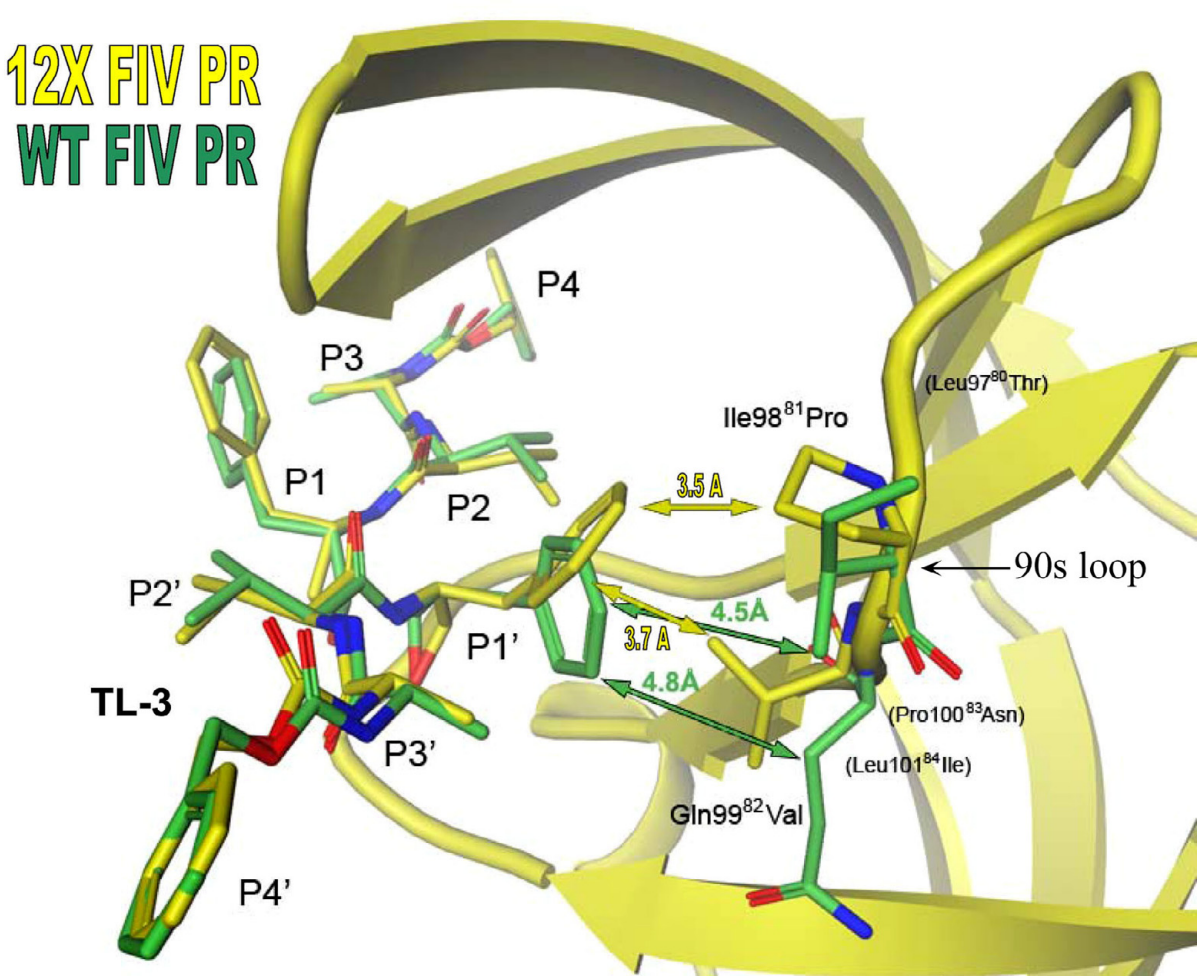
**Effects of the 90s loop mutations on interactions with TL-3**. Comparisons of the TL-3 complexes of wild-type FIV protease (green) and 12X protease (yellow) reveals conformational differences at the P1/P1' position of the inhibitor. The mutation of residue 98 from Isoleucine to Proline and residue 99 from Glutamine to Valine in the 12X mutant protease allows the formation of packing contacts with the P1/P1' position of TL-3, causing the P1/P1' phenyl ring to shift toward the side chain of Proline 98 by 2.0Å and rotate by 21° about the χ^1 ^torsion angle. These movements are facilitated by other mutations in the 90s loop and active site core (see Fig. 4).

**Figure 4:**
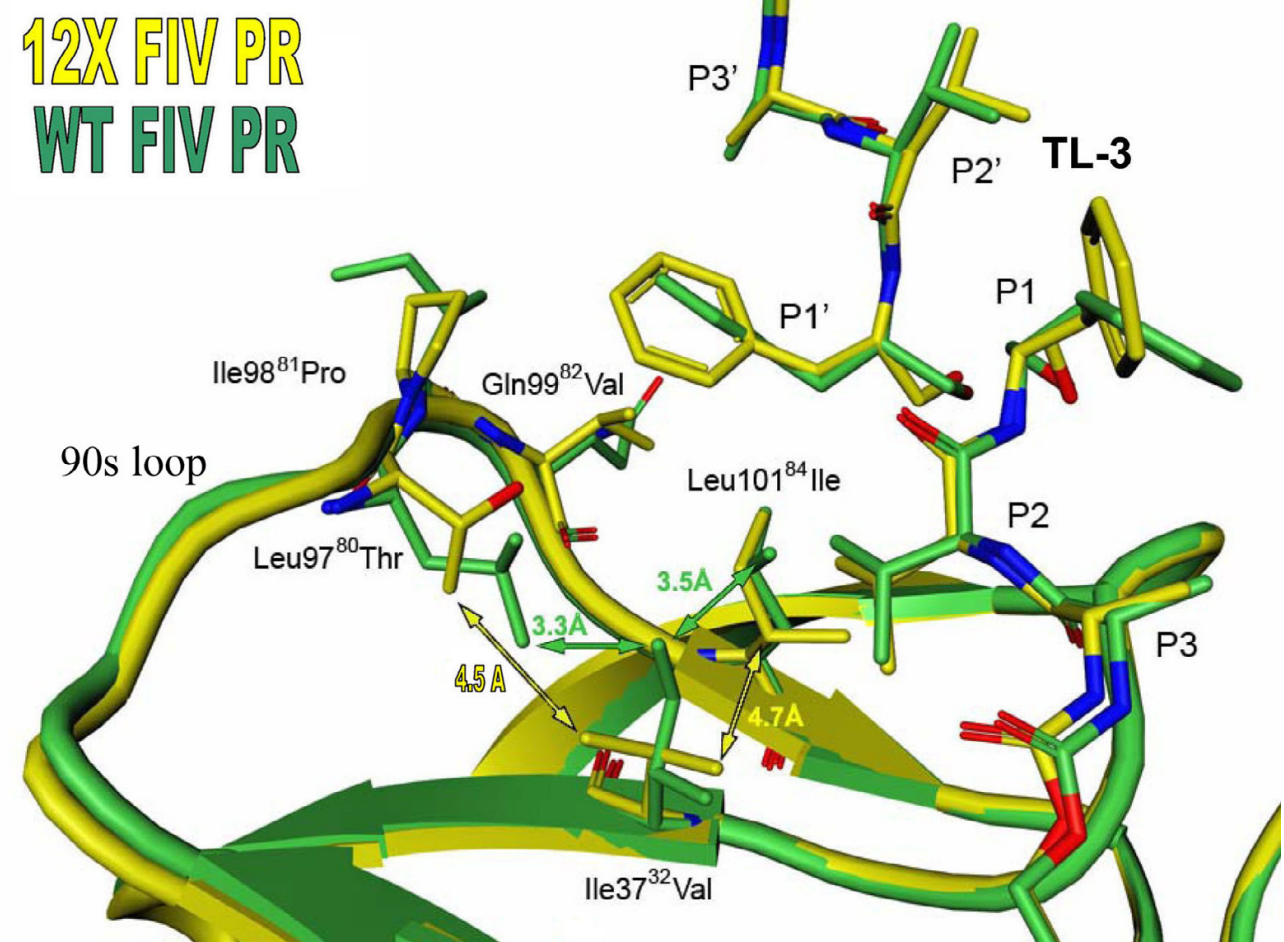
**Changes in the packing contacts between the active site core and 90s loop**. The reformation of the P1/P1' interaction of TL-3 and the 90s loop is aided by the loss of packing interactions between residue 37 in the active site and the 90s loop. In wild-type FIV protease (green) the side chain of Isoleucine 37 forms packing contacts with the side chains of Leucine 97 and Leucine 101, holding the 90s loop in position away from TL-3. The mutation of Isoleucine 37 to Valine, Leucine 97 to Threonine, and Leucine 101 to Isoleucine in the 12X mutant protease (yellow) eliminates these packing contacts, allowing the 90s loop to shift ~1.0Å toward the P1/P1' position of TL-3.

In HIV PR, the P1' phenyl ring of TL-3 is tightly packed against the side chains of Pro81 and Val82 in the "80s" loop of the two-fold related monomer [26]. In FIV PR, the structurally equivalent region spans residues 97 to 101 and is thus referred to here as the "90s loop". In this context, residues Ile98^81 ^and Gln99^82^, are positioned too far away to form van der Waals interactions with the P1' phenyl group of TL-3 (Fig. 3). Five residues in the 90s loop have been mutated to their corresponding HIV PR residues in the 12X FIV PR; these include Leu97^80^Thr, Ile98^81^Pro, Gln99^82^Val, Pro100^83^Asn and Leu101^84^Ile. In the 12X FIV PR complex with TL-3, the P1' phenyl group is again able to pack against the side chains of Pro98^81 ^and Val99^82 ^reforming important interactions between the protein and inhibitor (Fig. 3). The ability of the 90s loop to shift towards the bound TL-3 and reform this packing contact is facilitated by three additional mutations, Ile37^32^Val, Leu97^80^Thr and Leu101^84^Ile. In the WT FIV PR-TL-3 complex, the side chain of Ile37^32 ^forms packing contacts with the side chains of Leu97^80 ^and Leu101^84 ^which holds the 90s loop in position, away from the P1' subsite of TL-3 (Fig. 4). The mutation of Ile37^32 ^to Val, Leu97^80 ^to Thr, and Leu101^84 ^to Ile abolishes these packing contacts, allowing the 90s loop to shift toward the bound inhibitor, therefore promoting the reformation of the packing contacts between the P1' phenyl group of TL-3, Pro98^81 ^and Val99^82^. Hence, the "HIVinizing" replacements affect TL-3 binding directly, and indirectly, as a consequence of buried side chain interactions. Restoration of the packing interactions increases the inhibition by TL-3 relative to wild-type FIV PR by a factor of 3.7 (K_i_^12X FIV PR ^= 10 nM; K_i_^WT FIV PR ^= 41 nM) [2-4]. However, TL-3 inhibition remains over 7-fold weaker relative to wild-type HIV PR (K_i_^HIV PR ^= 1.5 nM).

### Intra-flap and inter-flap interactions stabilize the closed conformation of the flap regions in 12X FIV PR

**Figure 5:**
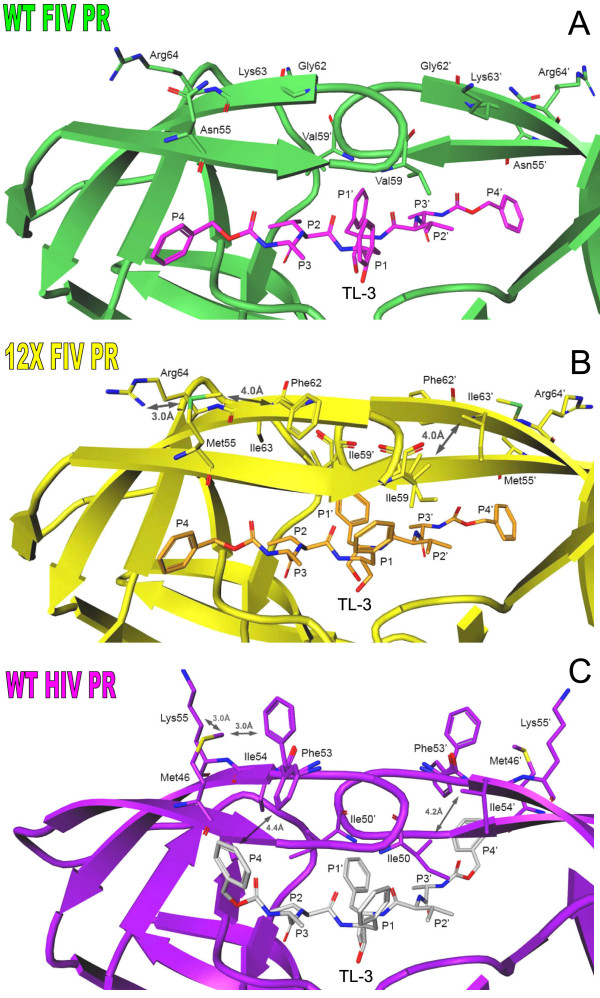
**Comparison of the flap regions of wild-type FIV protease, 12X FIV protease, and wild-type HIV protease**. **(a) **In the wild-type FIV protease, residues positioned at the top and tips of the flaps are not able to form stabilizing interactions. **(b) **In the 12X mutant Asparagine 55 has been mutated to Methionine and Glycine 62 has been mutated to Phenylalanine, allowing the formation of an intra-flap packing contact between these two residues and an electrostatic interaction between S^δ ^of Methionine 55 and N^η ^of Arginine 64. Two additional substitutions in the flap regions of 12X FIV protease, Valine 59 to Isoleucine, and Lysine 63 to Isoleucine, result in the formation of an inter-flap packing contact between the isoleucines (Isoleucine 59 ... Isoleucine 63'). The introduction of stabilizing contacts due to these mutations increases the overall stability of the closed conformation of the flaps. **(c) **The stabilizing contacts formed as a result of the 12X flap mutations closely resemble those seen in the structure of wild-type HIV in complex with TL-3. The side chain of Methionine 46 is packed between the side chains of Phenylalanine 53 and Lysine 55 in the wild-type HIV protease, just as Methionine 55 is packed between the side chains of Phenylalanine 62 and Arginine 64 in 12X FIV protease (b). Also as in 12X FIV protease, an inter-flap packing contact is formed in HIV protease between Isoleucine 50 and Isoleucine 54'.

Six of the mutations introduced into 12X FIV PR are localized to the flap regions of the protein; Asn55^46^Met, Met56^47^Ile, Ile57^48^Gly, Val59^50^Ile, Gly62^53^Phe, Lys63^54^Ile (Fig. 1). Residues 55, 62 and 63 are positioned in the center of the flaps with their side chains pointing away from the active site (Fig. 5(a), (b)). The mutation of Asn55^46 ^to Met and Gly62^53 ^to Phe in 12X FIV PR results in the formation of two intra-flap interactions: a packing contact formed between the C^ε ^atom of Met55^46 ^and the side chain of Phe62^53^, and an electrostatic interaction between the S^δ ^atom of Met55^46 ^and the N^ε ^atom of Arg64^55 ^(Fig. 5(b)). This pair of intra-flap interactions closely mimics the pair of intra-flap packing contacts between Met46, Phe53 and Lys55 seen in the HIV PR-TL-3 complex structure (Fig. 5(c)) [13,26]. Additional mutations of Val59^50 ^to Ile and Lys63^54 ^to Ile result in the formation of flap interactions between monomers that is not present in wild-type FIV PR (Fig. 1). The introduction of the intra-flap and inter-flap interactions in 12X FIV PR may help to stabilize the closed conformation of the flap regions, and may be a contributing factor to the increased inhibition by TL-3. The stabilization of the flaps could increase the thermodynamic barrier to flap opening and, therefore affect substrate processing efficiency by increasing the residence time of substrate in the active site.

## Discussion

12X FIV PR is a transitional mutant with engineered drug susceptibility. The mutations found in 12X FIV PR change residues from their native amino acids to those at structurally equivalent positions in HIV PR. In this way, 12X FIV PR can be considered a transitional mutant that exhibits intermediate susceptibility to TL-3 (K_i_^WT FIV PR ^= 41 nM; K_i_^12X FIV PR ^= 10 nM; K_i_^HIV PR ^= 1.5 nM). While the 12 substitutions have no affect on the hydrogen bonding pattern between the protein and inhibitor, they do affect the packing interactions. The 90s loop in 12X FIV PR more closely resembles the 80s loop of HIV PR in sequence and conformational flexibility. The removal of a packing contacts formed by Val37^32^, Thr97^80 ^and Ile101^84 ^allows the 90s loop to shift more closely to the bound inhibitor. With the additional mutations of Ile98^81 ^to Pro and Gln99^82 ^to Val, the 90s loop becomes able to form the packing interaction with the P1' phenyl group of TL-3 as seen in the complex between HIV PR and TL-3. The loss of this particular packing contact was previously reported to result in a nearly 4-fold decrease in inhibition by TL-3 in 1X HIV PR, where 1X represents the V82A mutant (IC_50_^WTHIVPR ^= 6 nM; IC_50_^1X HIV PR ^= 22 nM) [1,26]. Hence, it is reasonable that recovery of this interaction in the 12X FIV PR would have the opposite affect, contributing to the TL-3 susceptibility of the enzyme (IC_50_^WT FIV PR ^= 90 nM; IC_50_^12X FIV PR ^= 71 nM).

The above findings account for observed changes in inhibitor specificity in the HIV/FIV chimeric PRs and support the involvement of targeted residues in the hinge, flap, and 90s loop in inhibitor binding (Fig. 1). Interestingly, changes in substrate cleavage are harder to institute, so that the virus is able to develop inhibitor resistance while replicating sufficiently to maintain virus production. As many as 24 HIV amino acid substitutions have been made in the FIV PR background without substantially increasing HIV substrate cleavage [17]. Mutations that increase stability in the flap allow a degree of cleavage of HIV substrates by FIV, but levels do not approach that obtained by HIV PR [17]. Several of the mutations in the 12X FIV PR could affect the stability of the flaps in either the open or closed state. In addition to Met55^46 ^and Phe62^53^, which stabilize individual flaps (Fig. 5b), Ile59^50 ^and Ile63^54 ^could form reciprocal packing interactions between flaps, favoring a closed conformation [27]. Loss of an equivalent interaction results in a 6 Å separation between flaps in the Phe53Leu mutant of apo-HIV PR [28]. Two of the flap residues replaced in 12X FIV PR (Asn55^46^Met, Lys63^54^Ile) are also sites of mutation in the 'wide-open' conformation of a multidrug-resistant HIV PR [29]. Molecular dynamics studies suggest that hydrophobic clustering of Val37^32^, Ile59^50^, Pro98^81^, and Val99^82 ^within monomers could stabilize an open conformation of the enzyme [27]. Saturation mutagenesis of HIV PR shows that of six flap residues mutated in 12X FIV PR, four (Met 55^46^, Gly57^48^, Ile59^50^, and Phe62^53^) result in intermediate activity if inserted into HIV PR, and two (Ile56^47 ^and Ile63^54^) inactivate the enzyme [30]. Clearly, the overall character of the PR contributes to the observed substrate specificity with the conformational preferences of the flaps being critical.

In addition, the context of PR in the natural substrate has a direct impact on overall processing efficiency. The critical role for PR in the virus life cycle is not only to process the Gag and Gag-Pol proteins specifically [31,32], but also to perform cleavages in the proper order and temporal sequence [33]. The processing sequence and efficiency of the HIV-1 Gag-Pol polyprotein has been studied in great detail and has been shown to be critical to generate infectious virus [20,33,34]. Of note is the finding that proper temporal cleavage of the Gag-Pol polyprotein is influenced by conformational constraints on PR "embedded" in the context of the polyprotein such that minor amino acid changes can alter the order of polyprotein cleavage [35]. In particular, the replacement P1A appears to enhance mobility of the dimeric, embedded protease [21,35]. Recent studies of FIV using the 12X mutant and additional FIV/HIV PR chimeras, when placed in the context of the Gag and Gag-Pol polyprotein, are consistent with the findings in HIV PR [4]. The results show that the chimeric PRs cleave the natural Gag polyprotein substrate expressed in the context of pseudovirions. However, the addition of HIV residues with concomitant increase in HIV character results in inappropriate order of cleavage [4]. Specifically, the NC-p2 cleavage junction was processed efficiently by wild type FIV PR, but poorly by the "HIVinized" FIV mutants. The junctions on either side of NC are the earliest processing sites and the proper timing of these cleavages is critical to generation of infectious HIV virions [20,21,34]. FIVs encoding the chimeric PRs are non-infectious and it is probable that temporal changes in processing are responsible, due to altered rates of cleavage arising from the structural changes identified here. Increased rigidity of the flaps of HIV PR has been previously demonstrated to alter substrate cleavage kinetics by increasing the off-rate [36]. Recent molecular dynamics simulations have emphasized the importance of flap mobility on function in the crowded molecular environment of the cell [37]. The phenomenon has also been observed in other systems where allosteric effects have led to an increased residency time in the enzyme active site [38,39]. Obtaining the structure of PR in the context of the polyprotein would be of great interest in better defining structural constraints, and stands as a challenge for future experimentation.

## Conclusion

The 1.7 Å resolution crystal structure of FIV protease (PR), in which 12 critical residues around the active site have been substituted with structurally equivalent residues in HIV PR, was determined in complex with the broad-based inhibitor TL-3. The structure, in comparison with structures of HIV and FIV PRs with TL-3 bound, demonstrates how substitutions which make FIV PR more HIV-like result in altered inhibition constants in the order HIV PR > 12X FIV PR > FIV PR. The analysis shows how 12X FIV PR gains several stabilizing intra- and inter-flap interactions that resemble those in HIV PR, while retaining hydrogen bonding interactions common to both FIV and HIV PRs. The structural details suggest that changes in flap mobility may be related to changes in substrate processing efficiency, thereby affecting cleavage of Gag and Gag-Pol sites by FIV *vs*. HIV protease. The results provide better understanding of the molecular basis of HIV-1 and FIV protease (PR) substrate specificities *in vivo*, and are relevant to the development of broad-spectrum protease inhibitors that can inhibit both wild type and drug-resistant proteases.

## Methods

### Mutagenesis of chimeric FIV PRs

Chimeric FIV PRs were constructed by substituting the residues of FIV PR for the structurally equivalent residues of HIV-1 PR with PCR-mediated megaprimer site-directed mutagenesis as described [17]. The chimeric PR genes were digested with *Nde*I and *Hind*III and cloned into pET-21a (Novagen, Inc.). The substitutions were verified by dideoxy DNA sequencing. All protease constructs were over-expressed in *E. coli *strain BL21.DE3/pLysS using T7-driven expression in the context of the pET21 vector (Novagen) [13,17]. Expression was induced by treatment of late log phase cells with 1 mM isopropylthiogalactopyranoside (IPTG) for 3 hr at 37°C.

### Purification and refolding of mutant FIV PR

PRs were purified and re-folded for crystallization following the previously described procedure [2]. Inclusion bodies containing 12X protease were purified by resuspending the cell pellet from 1 liter of cell culture in 20 mM Tris, 2 mM EDTA (TE), pH 8 buffer containing 1% NP-40 and stirring for 20 min at RT. The solution was then treated in a Waring blender for 30 seconds, and 100 ml of 8 M urea + TE buffer was added with stirring at 4 deg C for 20 min. Inclusion bodies were pelleted at 8,000 × g for 1 hr. and subsequently washed with deionized water until the pelleted inclusion bodies stuck to the side of the centrifuge tube (typically after the third wash). Inclusion bodies were solubilized in 8 M urea in TE buffer, 10 mM DTT with gentle rocking overnight at 4°C. Insoluble material was removed by centrifugation, followed by filtration through a 0.45 μm membrane. Solid DE52 (Whatman; 20 g) was then added and the solution was incubated at 4°C for 1 hr. and then filtered through a 0.45 μm membrane. The DE52 was discarded and the filtered solution containing protease was then applied to an RQ column (J.T. Baker) that had been equilibrated in 8 M urea, 20 mM Tris, 2 mM EDTA, pH 8.0. The column flow through containing the protease was collected and refolded by dialysis against 20 mM sodium phosphate, pH 7.2, 25 mM NaCl, and 0.2% 2-mercaptoethanol overnight at 4°C, followed by dialysis against 10 mM sodium acetate, pH 5.2, 0.2% 2-mercaptoethanol for 3 hr. The refolded protease was centrifuged for 20 min. at 38,000 g at 4°C to remove any precipitated material. The sample was then concentrated using a centrifuge concentrator (Amicon Ultra 10,000 MW cut-off), washed twice with 20 mM sodium acetate, pH 5.2 saturated with TL-3, and then concentrated to 5–10 mg/ml.

### Crystallization and data collection

#### Crystallization

1 μl of 12X FIV PR at 2.5 mg/ml with added TL-3 was mixed with 1 μl of 2.5 M lithium chloride, 100 mM Hepes, pH 7.5, and equilibrated by hanging drop vapor diffusion against this reservoir solution at 8°C. Prismatic trigonal crystals formed within one week. The crystals were transferred to a synthetic mother liquor solution containing 15% propylene glycol for a several seconds, and then flash frozen in liquid N_2_.

#### Data collection

Diffraction data were collected at 100 K by the rotation method (120 frames, 1° oscillation per frame) to 1.7 Å resolution at beam line 1–5 (λ = 0.979 Å) at the Stanford Synchrotron Radiation Laboratory. The data were processed with Mosflm [40] and Scala [41] (Table 1).

### Structure solution and refinement

The structure of 12X FIV protease was solved by molecular replacement at 3 Å resolution using coordinates of the monomer of wild-type FIV protease (PDB 1B11) as a search model in Molrep [42]. Residues differing in sequence between the two proteins were modeled as alanines. Five percent of randomly selected reflections were designated as test reflections for use in the Free-R cross-validation method [43] and used throughout the refinement. The correlation coefficient and R-factor from the molecular replacement solutions indicated that the correct space group was *P*3_1_21. Rigid body and restrained refinement were performed in Refmac [44] at 3 Å and 2.0 Å, respectively. Simulated annealing, Powell minimization and individual temperature factor refinements were performed using CNS [45]. After refinement, the model was adjusted and correct amino acids were built into regions of the composite omit map using the visualization program O [46]. The model was refined in CNS [45] using a bulk solvent correction and isotropic B-factors, followed by several rounds of model adjustment using the SigmaA-weighted 2|F_o_|-|F_c_| and |F_o_|-|F_c_| electron density maps [47] generated in CNS [45]. TL-3 was initially modeled by superposition of the wild-type FIV structure in complex with TL-3 (1B11). The conformation of the bound TL-3 was manually adjusted to fit the SigmaA-weighted |F_o_|-|F_c_| electron density (2σ). 121 water molecules were added and nine residues were model as having alternate side chain conformations. The region between Ile59 and Gly61 was modeled with two main chain conformations that contained a flipped peptide bond between Ile59 and Gly60. The model was refined to a final R_cryst_/R_free _of 18.4/23.3% [43,45] (Table 1).

**Table 1:** Crystallographic Statistics

*Unit Cell*		
Space group		P3_1_21
a, b, c (Å)		50.32 50.32 74.16
V_M _(Å^3^/Da)		2.5
Solvent content (%)		49.7
Monomers/asymmetric unit		1

*Data Collection*		

SSRL beam line		BL 1-5
Wavelength (Å)		0.979
Resolution range (Å)		74.0 – 1.70
Observations		77,552
Reflections		12,424
Redundancy		6.2 (6.0)
Completeness (%)^[1]^		99.7 (99.6)
<I>/<σ_I_>		15.7 (2.6)
Rsymm(I)^[2]^		0.070 (0.368)

*Refinement*		

Reflections > 0.0 σ_F_		12,396
R-factor^[3]^		0.184
Rfree (% of data)		0.233 (5.0)
R.m.s. deviation, bonds (Å)		0.011
R.m.s. deviation, angles (deg)^[4]^		1.47

*Model*		

*Protein*	*Atoms*	*<B-factor> (Å^2^)*
Protein^[5]^	1,134	21.2/24.6^[6]^
TL-3	33	19.8
Water molecules	121	38.5

[1] Values for highest resolution shell in parentheses.

[2] R_symm _= Σ_hkl_Σ_i_|I_i_(hkl) - I(hkl)|/Σ_hkl_Σ_i_(I(hkl)) where I_i_(hkl) is the intensity of an individual measurement, and I(hkl) is the mean intensity of this reflection.

[3] R-factor = Σ_hkl_|F_obs_| - |F_calc_|/Σ_hkl_|F_obs_|, where |F_obs_| and |F_calc_| are observed and calculated structure factor amplitudes, respectively.

[4] Ramachandran plot: 95.9% of residues in most favored regions; 3.1% in allowed regions, 1.0% in disfavored regions.

[5] Includes residues with alternate conformations.

[6] Average B-factors for main chain and side atoms, respectively.

### Protein Data Bank accession numbers

The 12X FIV protease complex crystal structure with the inhibitor TL-3 has been deposited into the RCSB Protein Data Bank and has been assigned the accession code 2HAH.

## Competing interests

The author(s) declare that they have no competing interests.

## Authors' contributions

YCL and KT prepared the protein samples, and HH grew the crystals and performed crystallographic analysis. BET and JHE developed the TL-3 inhibitor, and JHE directed the design of the 12X chimeric FIV protease. CDS supervised the structural analysis. All authors read and approved the final manuscript.
